# Vitamin A and risk of bladder cancer: a meta-analysis of epidemiological studies

**DOI:** 10.1186/1477-7819-12-130

**Published:** 2014-04-29

**Authors:** Jian-er Tang, Rong-jiang Wang, Huan Zhong, Bing Yu, Yu Chen

**Affiliations:** 1 Department of Urology, The First Affiliated Hospital, Huzhou Teachers College, Guangchanghou Road 158, Huzhou 313000, Zhejiang Province, China

**Keywords:** Bladder cancer, Meta-analysis, Retinol, Vitamin A

## Abstract

**Background:**

Epidemiological studies have reported the preventive effect of vitamin A intake on bladder cancer. However, the findings are inconsistent. To address this issue we conducted a meta-analysis to investigate the quantitative effects of vitamin A on bladder cancer.

**Methods:**

We searched MEDLINE and Embase databases and the references of the relevant articles in English to include studies on dietary or blood vitamin A for the risk of bladder cancer. We performed a meta-analysis using both fixed-effects and random-effects models.

**Results:**

Twenty-five articles on dietary vitamin A or blood vitamin A were included according to the eligibility criteria. The pooled risk estimates of bladder cancer were 0.82 (95% CI 0.65, 0.95) for total vitamin A intake, 0.88 (95% CI 0.73, 1.02) for retinol intake, and 0.64 (95% CI 0.38, 0.90) for blood retinol levels. We also found inverse associations between subtypes of carotenoids and bladder cancer risk.

**Conclusion:**

The findings of this meta-analysis indicate that high vitamin A intake was associated with a lower risk of bladder cancer. Larger studies with prospective design and rigorous methodology should be considered to validate the current findings.

## Background

Bladder cancer is the fifth most common cancer among with an estimated 386,000 new cases and 150,000 deaths world-wide in 2008 [1]. It has the highest lifetime treatment cost for any cancer [2]. Carcinogens or dietary chemopreventive agents can be concentrated in urine and have prolonged exposure to the bladder epithelium, making it an ideal target for preventative strategies [3].

Environmental factors, particularly dietary factors, have been postulated to play important roles in the etiology of bladder cancer [4]. Vitamin A and retinol are hypothesized to reduce the risk of bladder cancer due to their roles in the regulation of cell differentiation and apoptosis [5]. A previous meta-analysis, including seven case–control studies and three cohort studies, found no association of bladder cancer in relation with diets low in retinol and β-carotene [6]. Since the meta-analysis was published, epidemiologic studies of vitamin A, retinol (preformed vitamin A), and carotenoids in relation to the risk of bladder cancer have documented inconsistent results. In the present study, we analyzed this relationship further by conducting an updated meta-analysis of relevant studies. This updated analysis will allow us to provide more precise risk estimates than the previous analysis according to different carotenoids. We also examined the association between blood vitamin A concentrations and bladder cancer risk.

## Methods

### Search strategy

We searched MEDLINE and Embase databases to September 2013 for studies in humans on the relationship between vitamin A and incidence of bladder cancer. The search query was the following: (retinol or “vitamin A” or beta-carotene or carotenoids) and (“bladder cancer” or “urothelial cancer” or “urinary tract cancer” or “urinary bladder neoplasms” [MeSH Terms]). We also reviewed the reference lists from reviews, meta-analyses and other relevant publications to search for further studies to be included.

### Selection criteria

Studies were included in this meta-analysis if they met the following criteria: they presented original data from case–control or cohort studies; the exposure of interest was intake of vitamin A (retinol, carotene, or other carotenoids) or blood (plasma or serum) levels of vitamin A; the outcome of interest was bladder cancer or urothelial cancer; and odds ratio or relative risk estimates with 95% confidence intervals (CIs) (or data to calculate these) were reported and adjusted for at least age, sex, and smoking. If the same study was used in more than one publication, we included the one with the largest number of cases. Because the overwhelming majority of tumors occurred in the bladder, and the renal pelvis and ureter are covered by the same urothelium, the term bladder cancer was used as a synonym for all neoplasms of the bladder, renal pelvis, and ureter.

### Data extraction

Data extracted from each study were the following: the first author, publication year, country where the study was carried out, study period, participant sex and age, sample size, anatomical site of the neoplasm, research contents, study quality, and adjusted covariates. We used the odds ratio or relative risk with 95% CI of the highest intake (or blood level) group for bladder cancer compared with the lowest intake (or blood level) group reported in each study. Data extraction was conducted independently by two authors (JT and RW), with disagreements resolved by consensus.

### Quality assessment

We assessed the quality of all included studies using the Newcastle-Ottawa scale. (http://www.ohri.ca/programs/clinical_epidemiology/oxford.asp). This is an eight-item instrument that allows for the assessment of the patient population and selection, study comparability, follow-up, and the outcome of interest. Interpretation of the scale is performed by awarding points, or ‘stars’, for high-quality elements. The stars are then added up and used to compare study quality in a quantitative manner. The maximum score is nine points, representing the highest methodological quality.

### Statistical analysis

Depending on heterogeneity between studies, we used fixed- or random-effects models to calculate summary risk estimates (REs) and 95% CIs for the highest versus the lowest levels of vitamin A. Statistical heterogeneity among studies was evaluated using the Q [7] and *I*^*2*^[8] statistic. In the sensitivity analysis, one study at a time was removed and the rest analyzed to evaluate whether the results could have been affected significantly by a single study. We also conducted subgroup analyses by study design, sex, geographic area, and source of vitamin A intake (diet or supplement). If results were reported for both dietary and total vitamin A (foods and supplements combined), we used the results for total vitamin A in the main analysis. Publication bias was evaluated with the use of the Egger’s test [9]. All statistical analyses were carried out with Stata 11 (StataCorp, College Station, TX, USA). A two-tailed *P*-value of less than 0.05 was considered to be statistically significant.

## Results

**Figure 1:**
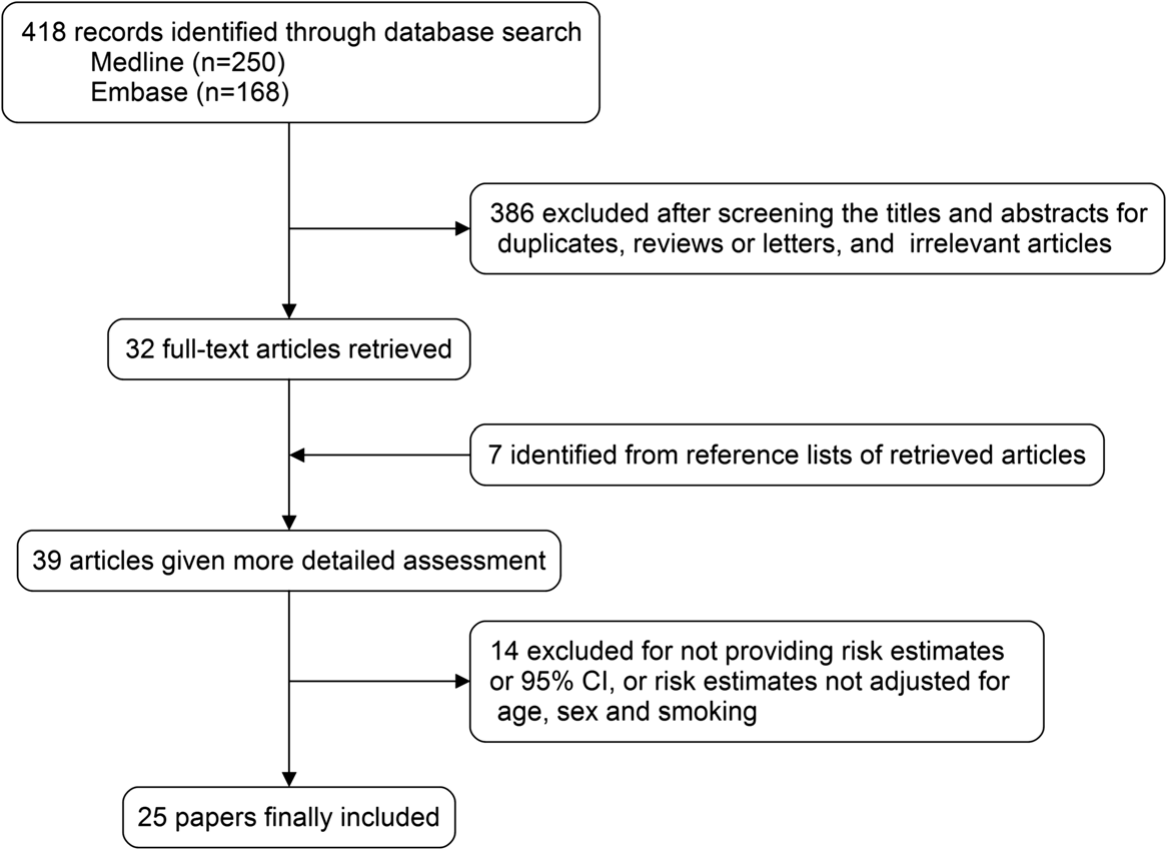
Flowchart of study selection.

A flowchart of the identification of relevant studies is shown in Figure 1. We identified a total of 418 articles (168 from Embase and 250 from MEDLINE) in our initial search. Of these, 386 were excluded after screening the titles and abstracts because of duplicates, reviews, and non-relatedness. Seven articles were identified from the references of relevant studies. The remaining 39 articles were given a more detailed assessment, and 14 studies were excluded because they did not meet the inclusion criteria. Finally, 25 papers were included in the present meta-analysis [10-34].

Table 1 shows the main characteristics of all 25 studies included in the final analysis. The 25 studies were published between 1988 and 2013 and involved a total of 11,580 cases. Eleven were cohort studies [14,16,18-20,23,25,29,31,32],[34] and 14 were case–control studies [10-13,15,17,21,22,24,26-28,30],[33]. Fifteen studies were conducted in North America [10,12,14-16,20-24,28,30,31,33],[34], eight in Europe [11,13,18,19,26,27,29,32], and two in Japan [17,25]. All 25 studies provided REs adjusted for at least age, sex, and smoking. Five reports provided results for blood vitamin A levels [20,24,25,28,32]; three measured plasma vitamin A levels [24,28,32] and two measured serum vitamin A levels [20,25]. Some studies included neoplasms of the urinary tract as cases [11,12,18,20,25,29,32], most of which were found to involve bladder cancer, whereas others selected only bladder cancer. The quality score of studies ranged from six stars to nine stars according to the nine-star Newcastle-Ottawa Scale except for one study by Hung *et al*. (five stars) [24].

**Table 1:** Study characteristics of published cohort and case–control studies on vitamin A and bladder cancer risk

**Authors and publication year**	**Study design**	**Country**	**Study period**	**Sex**	**Age (years)**	**Cases/subjects**	**Anatomical site**	**Parameters examined**	**Study quality**^**a**^	**Variables of adjustment**
[10]	PCC	Canada	1977 to 1982	M/F	35 to 79	826/1,618	Bladder	Diet: vitamin A, retinol, β-carotene	7	Age, sex, area of residence, smoking, history of diabetes
[11]	PCC	Sweden	1985 to 1987	M/F	40 to 74	418/929	Urothelium	Supplement: vitamin A, β-carotene	7	Age, sex, smoking
								Diet: retinol		
[12]	PCC	USA	1977 to 1986	M/F	30 to 93	261/783	Urothelium	Total: vitamin A, retinol, carotenoids	8	Age, sex, ethics, smoking
[13]	PCC	Spain	1985 to 1986	M	< 80	432/1,224	Bladder	Diet: retinol, carotene	8	Age, sex, smoking, total calories
[14]	Cohort	USA	1981 to 1989	M	65 to 84	71/70,159	Bladder	Supplement: vitamin A	5	Age and smoking
								Diet: β-carotene		
[15]	PCC	USA	1981 to 1984	M/F	45 to 65	262/667	Bladder	Diet: vitamin A, retinol, β-carotene	8	Age, sex, county, smoking, calories
								Supplement: vitamin A, retinol		
								Total: vitamin A		
Michand *et al*. 2000	Cohort	USA	1986 to 1998	M	40 to 75	320/51,529	Bladder	Total: vitamin A	7	Age, energy, pack-years of smoking, current smoking status, geographic region of the USA, cruciferous vegetable intake, total fluid intake
[17]	HCC	Japan	1996 to 1999	M/F	20 to 99	297/692	Bladder	Diet: vitamin A, retinol, carotene	6	Age, sex, smoking, occupational history as a cook
								Total: vitamin A, retinol, carotene		
[18]	Cohort	Netherlands	1986 to 1992	M/F	55 to 69	569/3,692	Urinary tract	Total: retinol, α-carotene, β-carotene, β-cryptoxanthin, lycopene, lutein/zeaxanthin	8	Age, sex, cigarette smoking amount, duration of smoking
Michand *et al*. 2002	Cohort	Finland	1985 to 1998	M	50 to 69	344/27,111	Bladder	Diet: vitamin A, α-carotene, β-carotene, β-cryptoxanthin, lycopene, lutein/zeaxanthin	7	Age, duration of smoking, smoking dose, total energy, trial intervention
[20]	Cohort (nested)	USA	1971 to 1995	M/F	52 to 71	111/222	Urothelium	Serum: retinol, carotenoids, α-carotene, β-carotene, β-cryptoxanthin, lycopene, lutein/zeaxanthin	8	Age, sex, smoking
[21]	PCC	USA	1987 to 1996	M/F	25 to 64	1,592/3,184	Bladder	Diet: retinol, carotenoids, α-carotene, β-carotene, β-cryptoxanthin, lycopene, lutein/zeaxanthin	8	Age, sex, education, number of cigarettes smoked per day, number of years of smoking, smoking status, lifetime use of non-steroidal anti-inflammatory drugs, number of years employed as a hairdresser/barber
[22]	HCC	USA	1999 to 2003	M/F	Not mentioned	409/860	Bladder	Diet: carotenoids, provitamin A, non-provitamin A	7	Age, gender, ethnicity, smoking status, pack-years of smoking, total energy
[23]	Cohort	USA	1980 to 2000	F	30 to 55	237/88,796	Bladder	Total: vitamin A, α-carotene, β-carotene, β-cryptoxanthin, lycopene, lutein/zeaxanthin	7	Age, pack-years of smoking, current smoking, total caloric intake
[25]	Cohort (nested)	Japan	1990 to 2007	M/F	>40	42/1,666	Urothelium	Serum: provitamin A, retinol, carotenes, carotenoids, α-carotene, β-carotene, β-cryptoxanthin, lycopene, lutein/zeaxanthin	7	Age, sex, smoking, alcohol consumption, body mass index, total cholesterol, education
[24]	HCC	USA	1993 to 1997	M/F	Not mentioned	84/257	Bladder	Plasma: α-carotene, β-carotene, β-cryptoxanthin, lycopene, lutein, zeaxanthin	5	Age, sex, pack-years of smoking, education
[26]	PCC	Belgium	1999 to 2004	M/F	Not mentioned	178/540	Bladder	Diet: retinol	7	Sex, age, smoking status, number of cigarettes smoked per day, number of years smoking, occupational exposure to PAH or aromatic amines, total fruit and vegetable consumption, intake of vitamin E and C and total anti-oxidant status
[27]	HCC	Spain	1998 to 2001	M/F	Not mentioned	912/1,789	Bladder	Diet: retinol and carotenoids	6	Age, gender, region, smoking status, smoking duration
[28]	HCC	USA	1999 to	M/F	Not mentioned	386/773	Bladder	Diet: retinol	6	Age, gender, ethnicity, smoking status, number of cigarettes per day, smoking duration
								Plasma: retinol		
[29]	Cohort	Denmark	1993 to 2006	M/F	50 to 64	322/55,557	Urothelium	Diet: β-carotene	8	Age, intake of vitamin C, vitamin E, and beta-carotene, smoking status, smoking duration, smoking intensity, passive smoking, work exposure.
								Supplement: β-carotene		
								Total: β-carotene		
[30]	PCC	USA	1997 to 2001	M/F	25 to 74	322/561	Bladder	Total: carotenoids, α-carotene, β-carotene, β-cryptoxanthin, lycopene, lutein	8	Age, sex, smoking status, total energy intake
[31]	Cohort	USA	2000 to 2007	M/F	50 to 76	330/77,050	Bladder	Supplement: β-carotene and retinol	8	Age, sex, race, education, family history of bladder cancer, smoking status/recency of smoking, pack-years of smoking, servings per day of fruits, servings per day of vegetables
[33]	PCC	USA	2001 to 2004	M/F	30 to 79	1,418/2,589	Bladder	Diet: vitamin A, α-carotene, β-carotene	8	Age, gender, region, race, Hispanic status, smoking status, usual body mass index, total energy
[32]	Cohort (nested)	Europe	1990 to 2005	M/F	25 to 70	856/1,712	Urothelium	Diet: β-carotene	8	Age at blood collection, study center, sex, date of blood collection, time of blood collection, fasting status; further adjusted for smoking status, duration, and intensity
								Plasma: carotenoids, α-carotene, β-carotene, β-cryptoxanthin, lycopene, lutein, zeaxanthin		
[34]	Cohort	USA	1993 to 2007	M/F	45 to 75	581/185,885	Bladder	Diet: vitamin A, α-carotene, β-carotene, β-cryptoxanthin, lycopene, lutein	8	Age, ethnicity, total energy intake, family history, employment in a high-risk industry, smoking, number of years since quitting, interactions of ethnicity with status

^a^Evaluated by the nine-star Newcastle-Ottawa Scale. HCC, hospital-based case–control study; PCC, population-based case–control study.

### High versus low vitamin A intake or blood vitamin A levels

**Figure 2:**
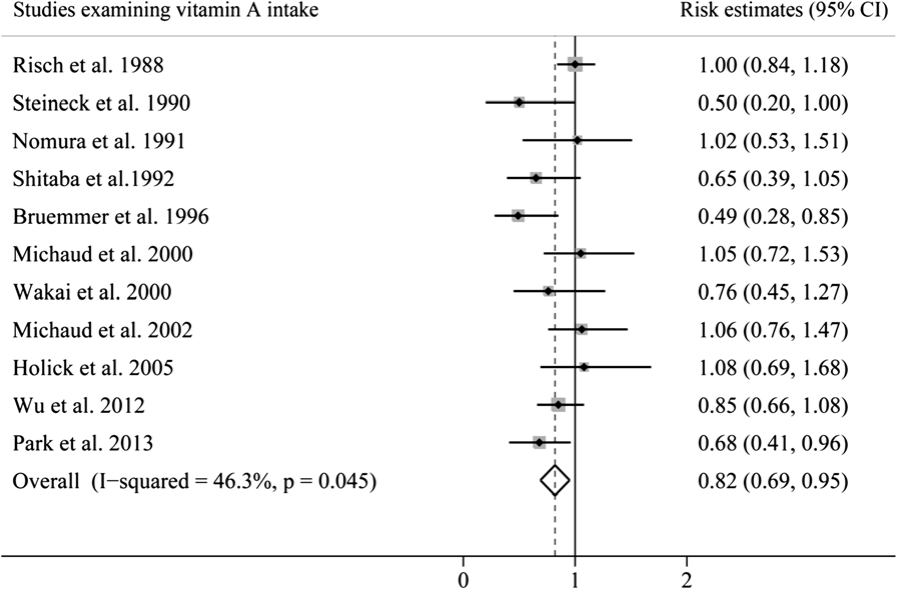
Pooled risk estimates of bladder cancer for the highest versus lowest categories of total vitamin A intake.

The analyses of vitamin A intake and bladder cancer risk were based on 11 studies. Figure 2 shows that results on vitamin A intake in relation to bladder cancer risk were inconsistent, with both inverse and positive associations reported. The pooled RE of bladder cancer for the highest versus lowest categories of vitamin A intake was 0.82 (95% CI 0.65, 0.95), suggesting that vitamin A intake was significantly associated with decreased risk of bladder cancer. There was moderate heterogeneity among studies (*P* = 0.045, *I*^*2*^ = 46.3%). The Egger test showed no evidence of publication bias (*P* = 0.057).

**Figure 3:**
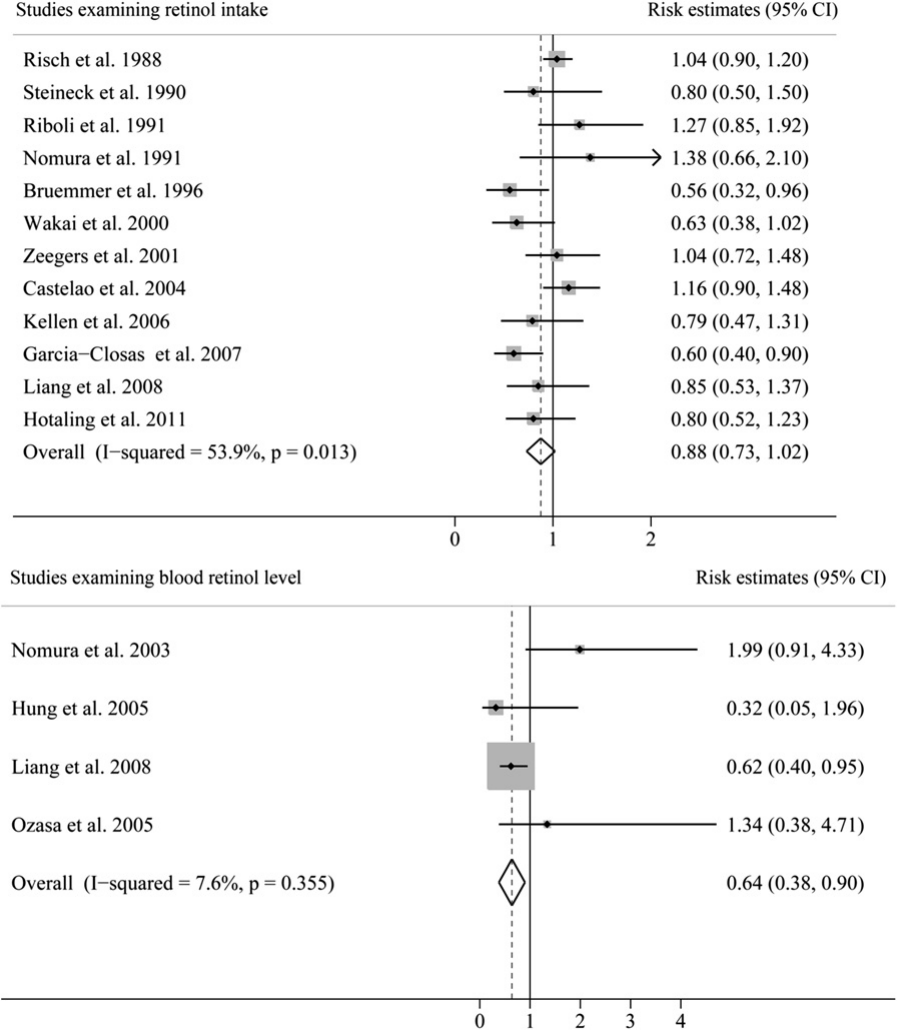
Pooled risk estimates of bladder cancer for the highest versus lowest categories of retinol intake or blood retinol level.

The REs for each study and all studies combined for the highest versus lowest categories of retinol intake or blood retinol level are shown in Figure 3. High intake of retinol was associated with a reduced but non-significant risk of bladder cancer (RE 0.88; 95% CI 0.73, 1.02), whereas high blood level of retinol was strongly associated with reduced risk of bladder cancer (RE 0.64; 95% CI 0.38, 0.90). There was significant heterogeneity among studies of retinol intake (*P* = 0.013; *I*^*2*^ = 53.9%) but not among studies of blood retinol levels (*P* = 0.355; *I*^*2*^ = 7.6%). The *P-*value for the Egger test of retinol intake was 0.10, suggesting a low probability of publication bias.

To explore the heterogeneity among studies of vitamin A intake and bladder cancer, we performed sensitivity analyses. A sensitivity analysis omitting one study at a time and calculating the pooled REs for the remaining studies showed that the study by Bruemmer *et al*. [15] substantially influenced the heterogeneity for total vitamin A intake. After excluding this single study, there was no study heterogeneity (*P* = 0.198; *I*^*2*^ = 26.7%), and the RE for the highest versus lowest category of vitamin A intake was essentially unchanged (RE 0.88; 95% CI 0.78, 0.97). However, we found no study significantly influenced the pooled RE for retinol intake.

We also performed subgroup analyses by study design, geographical region, gender, and source of intake (Table 2). For total vitamin A intake, when carrying out a stratified analysis for study design, the summary RE intake became non-significant for cohort studies. A significant inverse association was observed in North American studies, but not in European and Japanese studies. In a subgroup analysis by source of vitamin A intake, we observed a significantly decreased risk of bladder cancer in patients with supplementary intake, but not with dietary or dietary plus supplementary intake. For retinol intake, the stratified analysis did not show any statistically significant difference in summary estimates between strata. Unexpectedly, high intake of retinol in women seemed to increase the risk of bladder cancer significantly, although only two studies were included in this subgroup.

**Table 2:** Pooled risk estimates for the associations between total vitamin A and retinol and bladder cancer stratified by study design, gender, geographical region, and source of intake

**Subgroup**	**Total vitamin A**		**Retinol**	
	**RE (95% CI)**	***P***_**heterogeneity **_**(*I***^***2 ***^**score)**	**RE (95% CI)**	***P***_**heterogeneity **_**(*I***^***2 ***^**score)**
**Study design**				
Cohort	0.86 (0.67, 1.03)	0.200 (33.1%)	0.91 (0.65, 1.17)	0.366 (0)
Case–control	0.78 (0.59, 0.97)	0.027 (60.4%)	0.87 (0.70, 1.04)	0.006 (60.9%)
**Gender**				
Male	0.80 (0.66, 0.93)	0.160 (35.1%)	1.00 (0.69, 1.31)	0.067 (58.1%)
Female	0.68 (0.45, 0.91)	0.247 (27.6%)	1.51 (1.01, 2.00)	0.364 (0)
**Geographical region**				
Europe	0.79 (0.24, 1.34)	0.040 (76.3%)	0.65 (0.62, 1.09)	0.138 (42.7%)
US/Canada	0.83 (0.68, 0.98)	0.047 (50.8%)	0.94 (0.74, 1.13)	0.047 (55.5%)
Japan	0.76 (0.45, 1.27)	-	0.63 (0.38, 1.02)	-
**Source of intake**				
Diet	0.90 (0.80, 1.01)	0.426 (0)	0.87 (0.7, 1.04)	0.007 (60.3%)
Supplement	0.64 (0.47, 0.82)	0.568 (0)	0.80 (0.52, 1.23)	-
Diet and supplement	0.80 (0.49, 1.12)	0.045 (62.7%)	0.72 (0.43, 1.00)	0.110 (54.8%)

### High versus low carotenoids intake or blood carotenoids levels

The pooled REs of bladder cancer for the highest versus lowest categories of carotenoids intake or blood carotenoids levels are presented in Table 3. High intake of total carotenoids, α-carotene, β-carotene, and β-cryptoxanthin was associated with a significantly lower risk of bladder cancer. The summary REs of bladder cancer comparing the highest with the lowest category of intake were 0.67 (95% CI 0.55, 0.79) for total carotenoids, 0.87 (95% CI 0.76, 0.99) for α-carotene, 0.89 (95% CI 0.82, 0.97) for β-carotene and 0.86 (95% CI 0.73, 1.00) for β-cryptoxanthin. No significant associations were found between lutein and zeaxanthin, or lycopene intake and the risk of bladder cancer. There was a significant reduction in bladder cancer risk associated with increasing blood level of total carotenoids (RE 0.43; 95% CI 0.55, 0.79), α-carotene (RE 0.56; 95% CI 0.37, 0.75), β-carotene (RE 0.41; 95% CI 0.05, 0.78), and lutein and zeaxanthin (RE 0.50; 95% CI 0.12, 0.87), whereas no significant association was observed for β-cryptoxanthin or lycopene.

**Table 3:** Pooled risk estimates for the associations between carotenoids intake or blood carotenoids levels and bladder cancer risk

**Specific carotenoids**	**Number of studies**	**Pooled relative risk (95% CI)**	**Q-test for heterogeneity *P *** **value (*I***^***2 ***^**score)**
**Carotenoids intake**			
Total carotenoids	4	0.67 (0.55, 0.79)	0.524 (0)
α-carotene	8	0.87 (0.76, 0.99)	0.221 (27.2%)
β-carotene	12	0.89 (0.82, 0.97)	0.084 (38.6%)
β-cryptoxanthin	6	0.86 (0.73, 1.00)	0.427(0)
Lutein/zeaxanthin	6	0.93 (0.70, 1.17)	0.035 (58.2%)
Lycopene	6	0.95 (0.82, 1.07)	0.54 (0)
**Blood carotenoids level**			
Total carotenoids	2	0.43 (0.20, 0.93)	0.241 (27.3%)
α-carotene	4	0.56 (0.37, 0.75)	0.106 (51.0%)
β-carotene	4	0.41 (0.05, 0.78)	0.013 (72.4%)
β-cryptoxanthin	4	0.62 (0.06, 1.19)	0.027 (67.4%)
Lutein/zeaxanthin	4	0.50 (0.12, 0.87)	0.056 (50.2%)
Lycopene	4	0.60 (0.17, 1.03)	0.053 (61.0%)

## Discussion

Although vitamin A is found in a wide variety of foods, many people do not obtain an adequate intake of this nutrient. Therefore, the impact of vitamin A intake on bladder cancer risk has important public health implications Our findings were inconsistent with the previous meta-analysis [6], which suggested that no increased risk of bladder cancer were found for diets low in retinol (RE 1.01; 95% CI 0.83, 1.23) or beta-carotene (RE 1.10; 95% CI 0.93, 1.30) intake. We conducted an updated meta-analysis with results from new epidemiological studies presented in the past 13 years, and excluded results not adjusted for age, sex, and smoking. We complemented this analysis with pooled analyses of each carotenoid and blood vitamin A levels. The present meta-analysis suggests that increased vitamin A intake is associated with a reduced risk of bladder cancer. Retinol intake had a weak, but non-significant inverse association with the risk of bladder cancer, and carotenoids intake had a strong inverse association with the risk of bladder cancer. Moreover, both the blood total retinol and carotenoids levels had a significant association with the reduced risk of bladder cancer.

A preventive role of vitamin A in the development of bladder cancer is plausible. Retinol, the physiologically active form of vitamin A, and its metabolites (retinoids) play an important role in cell proliferation and differentiation [35]. Synthetic retinoids are effective in the prevention of bladder carcinogenesis in experimental animals [36]. We found that high retinol intake was associated with a borderline significant reduced risk of bladder cancer, and the non-significance might be attributed to the heterogeneity among studies. The anticarcinogenic properties of carotenoids are associated with their antioxidant activities; their modulation of carcinogen metabolism; their effects on cell translation and differentiation, cell-to-cell communication and immune function; and their inhibition of cell proliferation, oncogene expression and the endogenous formation of carcinogens [37]. While some carotenoids have potential to form vitamin A (provitamin A carotenoids, including α-carotene, β-carotene, and β-cryptoxanthin), others do not have this capability (non-provitamin A carotenoids such as lycopene, lutein, and zeaxanthin) [38]. Our results showed a significantly reduced risk of bladder cancer with high intake of α-carotene, β-carotene, and β-cryptoxanthin, but no association with lycopene, lutein, and zeaxanthin, suggesting that provitamin As are responsible for the chemoprotective effects of vitamin A.

Interestingly, we found that the inverse associations between vitamin A and risk of bladder cancer were more evident in blood levels. This may be because of measurement error in the assessment of vitamin A intake from food frequency questionnaires, leading to an attenuation of the observed association. By measuring vitamin A in blood, researchers are able to estimate the internal doses of nutrients. However, blood level of vitamin A only reflects a short-term dietary intake because the half-life of vitamin A in blood is only a few days. In addition, many people make changes to their diet by increasing the intake of dietary vitamin A after a cancer diagnosis as a way of staying as healthy as possible, and blood levels of vitamin A should be higher than before the diagnosis. This would underestimate the true associations of vitamin A with bladder cancer and result in bias of the RE toward the null. Despite this potential limitation, we found strong inverse associations between blood levels of retinol and some carotenoids and bladder cancer, which indicated that our estimates are relatively conservative.

Heterogeneity is often a concern in a meta-analysis, and we found a significant heterogeneity for total vitamin A and retinol intake. This may be due to the inherent methodological differences, such as study design and different ranges of exposure among studies. Although most studies adjusted for known risk factors for bladder cancer (age, sex, and smoking), residual or unknown confounding cannot be excluded as a potential explanation for the observed heterogeneity. Also, some studies measured vitamin A intake from diet only, whereas other studies combined dietary and supplemental vitamin A intake. We used a random-effects model to try to mitigate the heterogeneity as an issue, and our sensitivity analyses did not change the results for total vitamin A and retinol intake. We further performed stratified analyses by study design, sex, geographical region, and source of intake. Nevertheless, results were similar for total vitamin A and retinol intake throughout the subgroups.

A major strength of our study is the large number of included participants (11,580), and this is the first report to pool REs of bladder cancer for each carotenoid. In addition, our results were based on the adjusted evaluation. However, several limitations should be mentioned. First, as a meta-analysis of epidemiological studies, a food-frequency questionnaire was used to estimate the vitamin A intake, which may be influenced by the recall and information bias. Second, the sample sizes for several strata in the subgroup analyses were relatively small, and these results should be interpreted with caution. Finally, publication bias could be of concern because small studies with null results tend not to be published, though we found no evidence of publication bias in the meta-analysis.

## Conclusion

Our results support the hypothesis that diets high in vitamin A intake decrease the risk of bladder cancer. However, given these limitations and the heterogeneity of this meta-analysis, it is premature to recommend higher dietary vitamin A for the primary prevention of bladder cancer. Further investigation using large samples and a rigorous methodology is warranted.

## Abbreviations

CI: confidence interval; RE: risk estimate.

## Competing interests

The authors declare that they have no competing interests.

## Authors’ contributions

JT and RW wrote the manuscript; HZ and BY performed literature search and analyzed the data; YC performed statistical analysis; JT revised and edited the manuscript. All authors read and approved the final manuscript.
